# Antioxidant and antimicrobial effect of sodium alginate nanoemulsion coating enriched with oregano essential oil (*Origanum vulgare* L.) and *Trachyspermum ammi* oil (*Carum cupticum*) on food pathogenic bacteria

**DOI:** 10.1002/fsn3.3979

**Published:** 2024-02-16

**Authors:** Hashem Saffarian, Ebrahim Rahimi, Faham Khamesipour, Seyed Majid Hashemi Dehkordi

**Affiliations:** ^1^ Department of Food Hygiene and Public Health, Faculty of Veterinary Medicine, Shahrekord Branch Islamic Azad University Shahrekord Iran; ^2^ Faculty of Veterinary Medicine, Shahrekord Branch Islamic Azad University Shahrekord Iran; ^3^ Research Center for Hydatid Disease in Iran Kerman University of Medical Sciences Kerman Iran

**Keywords:** *Carum cupticum*, essential oil, food safety and antimicrobial resistance, *Origanum vulgare*

## Abstract

Today, microbial contamination in food is one of the major problems of the food industry and public health in general around the world. Foodborne illnesses, such as diarrheal diseases, kill many people around the world each year. The general objective of this study was to evaluate the antioxidant and antibacterial activity of sodium alginate nanoemulsion coating incorporated with oregano essential oil (*Origanum vulgare* L.) and *Trachyspermum ammi* oil (*Carum cupticum*) on *Escherichia coli*, and *Listeria monocytogenes*. To achieve this study, fresh chicken meat was used for this experiment. *Listeria monocytogenes* ATCC 19111 and *Escherichia coli* ATCC 35218 were obtained from the American Type Culture Collection (Manassas, VA, USA). After the preparation of the essential oil, the chemical composition of this essential oil was determined by using (GC–MS). The physicochemical properties of the nanoemulsion essential oil prepared were characterized and their antimicrobial activity was evaluated. The results showed that the GC–MS analysis of the volatile constituents of the *Origanum vulgare* essential oil compounds allowed the identification of 19 compounds representing 93.72% of the total oil. The major components detected in *Origanum vulgare* essential oil were pulegone (49.25%), eucalyptol (18.23%), and menthone (12.37%). About the *Carum cupticum* essential oil, 21 compounds representing 98.5% of the total oil were identified. The major components detected in *Origanum vulgare* essential oil were thymol (23.3%), p‐cymene (17.5%), and γ‐terpinene (16.8%). The best z‐average (d.nm) is 483.4 nm (*Carum cupticum* essential oil + nano) followed by 470.1 nm (nanochitosan). The results of the antimicrobial test showed that the different preparations have a good inhibitory activity for the in vitro growth of *Escherichia coli* and *Listeria monocytogenes*. According to the MIC and MBC results of this study, the nanoemulsion also presented a good bacteriostatic activity against the two pathogenic bacteria tested in this study.

## Graphical abstract

In this current study, the results showed that the physicochemical properties of EO nanoemulsion influenced by the effect of surfactant and the impact of ultrasonic emulsification for NE preparation, which have subsequently significant consequences on its antimicrobial activity. Hence, this essential oil and nanoemulsion exhibited antimicrobial activity against pathogenic bacteria. The results also demonstrated that nanoemulsion based on these plants has bacteriostatic and bactericidal activities against the food pathogenic bacteria.

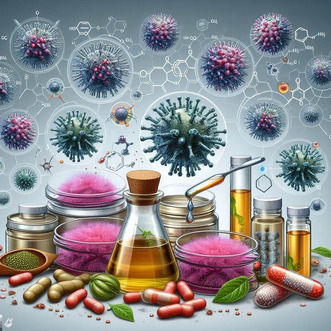

## 1 INTRODUCTION

The global population's health and well‐being face a significant risk due to the contamination of meals by microorganisms. This challenge stands out as one of the most critical issues confronting the food industry. Every year, food‐borne illnesses such as diarrhea are responsible for many deaths worldwide (Moghimi et al., 2016). The long‐standing use of synthetic antimicrobial preservatives and food additives (such as parabens) has delectable effects and sometimes alters the natural form of foods. The resulting economic losses are a major concern for consumers. This demonstrates the significance of using natural antibacterial agents or compounds derived from nature in the food sector. Reducing the level of synthetic antimicrobial compounds in food and other additives is necessary to maintain food safety (Enayatifard et al., 2021). The use of essential oils derived from plant sources as antibacterial additions is one natural option (Enayatifard et al., 2021).

Essential oils (EO), secondary metabolites derived from aromatic plants, are intricate blends of volatile and non‐volatile chemicals (da Silva et al., 2021). Due to their bioactive properties, they have been used since ancient times in various cultures, particularly in the food, pharmaceutical and cosmetics industries, thanks to their aromatic, antioxidant, and antimicrobial properties. EOs have been reported to have antibacterial (Nazzaro et al., 2013), antifungal (D'agostino et al., 2019), antiviral, and antioxidant effects. In the food sector, the use of nanotechnology is not yet too advanced but is increasingly attracting the interest of the scientific and industrial community. Recently, emulsions with small droplets, typically 10–100 nm in size, also known as nanoemulsions, are being studied as lipophilic drug delivery systems in food, cosmetic, and pharmaceutical goods (Enayatifard et al., 2021). Based on their intrinsic properties, there are several advantages for encapsulating functional lipophilic compounds over conventional emulsions.

*Origanum vulgare* L., a plant belonging to the *Lamiaceae* family, commonly known as oregano, is a safe, annual, perennial, and shrubby aromatic herb. It is utilized for therapeutic purposes all over the world in indigenous medicine for conditions such as dental caries, indigestion, respiratory issues, rheumatoid arthritis, and urinary tract disorders. The plant is widespread throughout Asia, particularly in Iran. Literature data have revealed that the plant exhibits antimutagenic, antioxidant, antihyperglycemic, antifungal, antiviral, anti‐inflammatory, and antibacterial effects. The essential oil extracted from this plant possesses antioxidant capacity and antimicrobial effects attributed to the presence of terpenoids and phenolic compounds such as thymol, carvacrol, γ‐terpinene, p‐cymene, sabinene, caryophyllene, germacrene, and spathulenol. Numerous studies have demonstrated the efficacy of oregano essential oil as a natural antimicrobial agent against various foodborne pathogens, including *Salmonella*, *Escherichia coli*, and *Listeria monocytogenes* (Burt, 2004; Leyva‐López et al., 2017). These antimicrobial effects are attributed to the presence of bioactive compounds such as carvacrol and thymol, which exhibit potent antibacterial and antifungal activities (Nazzaro et al., 2013). Incorporating oregano extracts or essential oil into meat products has shown promise in extending shelf life and enhancing safety by inhibiting the growth of spoilage and pathogenic microorganisms (Leyva‐López et al., 2017; Soultos et al., 2009). It has also been reported that the essential oil of *Trachyspermum ammi* (TAEO) is used for therapeutic purposes. *Trachyspermum ammi* is a plant with white flowers, reaching a height of 30–70 cm. It is grown in Afghanistan, Iran, and India (Khan and Jameel, 2018). Its medicinal value is linked to its organoleptic qualities and composition, which include thymol, γ‐terpinene, and para‐cymene (Kaur, 2017; Khan & Jameel, 2018). Recognized as natural compounds, these plants can be used as food additives in edible coatings to enhance food safety and prolong the shelf life of the food.

The limited solubility of essential oils in water is a technological limitation in the industry, necessitating innovative formulation processes. Nanoemulsions, which are very small droplet‐size emulsions, are innovative nanoscale drug delivery systems for bioactive components. This application would be useful for the preservation of food products. Furthermore, recent data have suggested that an improvement in the physical properties of essential oil‐loaded nanoemulsions compared to their typical similar emulsions and the use of nanoscale delivery systems can potentially increase passive cellular uptake mechanisms, thereby reducing resistance to mass transfer while increasing antimicrobial action. Additionally, higher antimicrobial activity was also noted in essential oil nanoemulsions. The potential of oregano as a natural preservative not only addresses concerns related to synthetic additives in the food industry but also aligns with the growing consumer demand for clean‐label and minimally processed products. As research in this area continues to unfold, exploring the practical applications and optimal concentrations of oregano extracts in meat preservation remains a focus for both scientists and the food industry. It is in this context that this study was initiated to evaluate the therapeutic effect of the sodium alginate nanoemulsion coating incorporated into the essential oil of oregano (*Origanum vulgare* L.) and the oil of *Trachyspermum ammi* (Carum cupticum). The overall goal of this study was to evaluate the antioxidant and antibacterial activity of the sodium alginate nanoemulsion coating infused with essential oil of oregano (*Origanum vulgare* L.) and oil of *Trachyspermum ammi* (Carum cupticum) on *Escherichia coli* and *Listeria monocytogenes*.

## 2 MATERIALS AND METHODS

### 2.1 Material

#### 2.1.1 Plant material and chicken meat

During this experimental study, fresh chicken meat was used as a biological sample. It served as an inoculation matrix for the bacteria used and stored in the refrigerator at 4°C. Sigma‐Aldrich (St. Louis, MO, USA) supplied food‐grade sodium alginate, glycerol, and Tween 80. Ultrapure water was utilized to prepare the coating solutions, The plant material used consisted of the organs of *Origanum vulgare* and *Trachyspermum ammi*. The leaves of the two plants were harvested and dried in the laboratory at 16°C away from light before being crushed. The resulting powder was treated to a hydrodistillation procedure utilizing an industrial‐type Clevenger apparatus for the distillation of essential oil (Özogul et al., 2022).

#### 2.1.2 Bacterial strains and cultural conditions

*Listeria monocytogenes* ATCC 19111 and *Escherichia coli* ATCC 35218 were obtained from the American Type Culture Collection (Manassas, VA, USA). All strains were maintained at −18°C in Tryptic Soy Broth (TSB; Merck, Darmstadt, Germany) containing 15% glycerol. The *L. monocytogenes* strain was cultured for 24 h at 37°C in TSB supplemented with 5 g/L yeast extract (TSBYE; Merck, Darmstadt, Germany). *Escherichia coli* was grown at Mueller Hinton culture broth for 24 h at 37°C.

### 2.2 Methods

#### 2.2.1 Preparation of the essential oil

According to the methodology described in the current European Pharmacopeia 6.0 (2008), dried bay leaves (10 kg) with a moisture content of 7% were subjected to a hydrodistillation process using an industrial Clevenger‐type apparatus for 4 h. The collected EO was then dried over anhydrous sodium sulphate (Na_2_SO_4_), filtered and stored in a light‐protected refrigerator at 4°C for further use.

#### 2.2.2 Chemical composition of this essential oil by using (GC–MS)

Gas chromatography–mass spectrometry (GC–MS) (Waltham, MA, USA) was used for the phytochemical characterization of essential oils. It was instantly connected to the mass spectrometer system (Perkin Elmer Clarus). The investigation was carried out on an SGE‐fused silica nonpolar capillary column (60 m × 0.25 mm, ID‐BPX5, 0.25 μm, Perkin Elmer, Shelton, CT, USA). To accomplish this, the oven program was set to 60°C for 10 min, then 250°C at a rate of 4 C/min for 10 min, and the final temperature was maintained for 10 min. The temperatures of the injector and detector were set at 220°C. At a flow rate of 1.5 mL/min, helium was used as the carrier gas. In spitless mode, 1 L of hexane‐diluted oil was injected. The temperature of the ion source was raised to 230°C with an electron ionization energy of 70 eV. The scan mass range was increased from 40 to 550 and the interface line temperature was maintained at 250°C. The identification of EO compounds was based on a comparison of their mass spectra with data from the NIST‐MS and WILEY‐MS libraries, as well as those published in the literature (Özogul et al., 2022).

#### 2.2.3 Preparation of oregano essential oil and *Trachyspermum ammi* oil nanoemulsion

The nanoemulsion based on the essential oil of these plants (oil‐in‐water) was created using a high‐energy process: ultrasonic emulsification as described by Ozogul et al. (2020), with minor modifications. The nanoemulsion (oil‐in‐water) was prepared using an oily phase constituting 11% of the total nanoemulsion and an aqueous phase consisting of 89%. It was prepared by mixing the essential oil of oregano and the essential oil of *Trachyspermum ammi* with Tween 80 (Sigma Aldrich, Taufkirchen, Germany) and water in a ratio of 10:1:89 w/w. The emulsions were then homogenized using an ultrasonic homogenizer (Optic Ivymen System CY‐500, Barcelona, Spain) for 15 min at 72 amplitudes. A sonotrode with a piezoelectric converter and a 14‐inch titanium alloy probe (5.6 mm diameter and 60 mm height) was used to provide energy input. The heat created by the emulsion was controlled throughout this procedure by wrapping ice around the beaker.

#### 2.2.4 Characterization of physicochemical properties of nanoemulsion essential oil

The polydispersity index (PDI) and average particle size of nanoemulsion droplets at 25°C were determined using a laser diffraction particle size analyzer (Mastersizer 2000 Malvern, UK) according to my methods published by Ozogul et al. (2020). Their thermodynamic stability was carried out according to the methodology of Shafiq et al. (2007). The viscosity of the nanoemulsion was determined using an ARES rheometer (Malvern Panalytical Ltd, Malvern, UK) at room temperature. The stability was tested using a centrifuge (at 2000 × *g* for 30 min) at 4 and 45°C. This procedure was repeated 6 times over 48 h. After that, tubes were kept at 21–25°C. NEs remained stable for up to 2 weeks. The refractive index was estimated by an Abbetype refractometer (Schmidt + Haensch ATR W2, Berlin, Germany).

#### 2.2.5 Antimicrobial activity

Before the antimicrobial test of the plant essential oil and nanoemulsion, Pencilin G, Vancomycin, Tetracycline, Chloramphenicol, Ciprofloxacin, Erythromycin, Gentamicin, Trimethoprim, Streptomycin, and Cephalexin were used for susceptibility test on the two strains bacteria. The paper disc diffusion method was applied for the determination of antimicrobial effects of oregano essential oil and *Trachyspermum ammi* oil‐in‐water nanoemulsion and pure oregano essential oil and *Trachyspermum ammi* essential oil against two pathogenic bacteria, and then the comparison between their activities (Yazgan et al., 2019), with slight modifications. In summary, 100 μL of bacterial suspension prepared on the 0.5 Mc Farland scale (108 CFU/mL) were spread on the surface of the solid agar medium contained in Petri dishes. Individually, sterile filter paper discs (6 mm in diameter) were impregnated with 50 μL of undiluted nanoemulsions and essential oils of oregano and *Trachyspermum ammi*, respectively. Then, the papers were separately deposited on the previously inoculated nutrient agars. Tetracycline (30 μg/mL), streptomycin (10 μg/mL), and neomycin (5 μg/mL) were used as positive controls and Tween 80 was used as negative control. The dishes were incubated at 37 ± 1°C for 18–24 h before measuring the diameters of the zones of inhibition surrounding the four discs were measured.

#### 2.2.6 Determination of minimum inhibitory (MIC) and minimum bactericidal concentration (MBC)

According to Yazgan et al. (2019), the bacteriostatic and bactericidal effects of nanoemulsion based on plant essential oil and pure essential oil against pathogenic bacteria were investigated using broth dilution methods. To do this, a 24‐h bacterial culture in broth was used. The final concentration of the microbial suspensions used was 1.106 CFU/mL. About 1 mL aliquots of pure, herbal essential oil were added separately to the first tube of each series and then serially diluted twice with Mueller Hinton Broth (MHB) to reach final concentrations. Subsequently, the inoculum suspension (1 mL) of each bacterial strain was added to each tube. A tube containing MHB and a bacterial suspension without emulsions or EO was used as a positive control, while a tube containing a nanoemulsion, EO, or Tween 80 with a bacterial suspension was utilized as a negative control. The tubes were then incubated at 37°C for 18 to 24 h with vigorous shaking. The MIC corresponded to the lowest concentration for which there was no microbial growth. Regarding the determination of the minimum bactericidal concentration (MBC), a culture of the content of the MICs was carried out on Mueller Hinton (MH) agar. The minimum concentration of samples, where no growth was observed after 24 h of incubation, was considered as MBC.

### 2.3 Statistical analysis

All tests were carried out in triplicate and the average value and standard deviation of the three measurements were reported. Duncan's multiple comparison test with SPSS version 19.0 for Windows (SPSS Inc., Chicago, IL, USA) was used to measure the significance of differences (*p* < .05).

## 3 RESULTS

### 3.1 Chemical composition of the plant essential oil

The results of the analysis of the phytochemical composition of the essential oil of *Origanum vulgare* by mass spectrophotometry are presented in Table 1. From the analysis of the results of this table, it appears that the essential oil of *Origanum vulgare* has 19 different compounds representing 93.72% of the total oil. The main components detected are pulegone (49.25%), eucalyptol (18.23%), and menthone (12.37%). The other components identified are in minute amounts ranging from 0.05% to 2.54%. We therefore deduce that the essential oil of *Origanum vulgare* is rich in bioactive composition.

**TABLE 1:** Chemical composition of *Origanum vulgare* essential oil analyzed by GC/MS.

Number	Chemical compound	R I	Percent (%)
1	α‐Pinene	934	1.42
2	Sabinene	975	1.52
3	β‐Pinene	981	2.54
4	Myrcene	992	0.61
5	3‐Octanol	1004	0.52
6	o‐Cymene	1031	0.14
7	Limonene	1034	0.86
8	Eucalyptol	1038	18.23
9	Linalool	1107	0.05
10	Menthone	1168	12.37
11	Isomenthone	1176	0.98
12	α‐Terpineol	1183	0.91
13	Terpinen‐4‐ol	1192	0.27
14	Pulegone	1254	49.5
15	Piperitone	1268	0.51
16	Piperitenone	1355	1.84
17	Caryophyllene	1427	0.35
18	β‐Farnesene	1456	0.13
19	Caryophyllene oxide	1596	0.97
	Total		93.72

The components of *Carum cupticum* essential oil analyzed identified are given in Table 2. The GC–MS analysis of the volatile constituents of the compounds of the essential oil of *Carum cupticum* allowed the identification of 21 compounds representing 98.5% of the total oil. The main components identified are thymol (23.3%), p‐cymene (17.5%), and γ‐terpinene (16.8%). The percentage of other phytochemical constituents varies from 1% to 5.7%. *Carum cupticum* essential oil is also rich in bioactive phytochemicals.

**TABLE 2:** Chemical composition of *Carum cupticum* essential oil analyzed by GC/MS.

Number	Chemical compound	R I	Percent (%)
1	α‐Thujene	936	2.9
2	α‐Pinene	948	1.1
3	Sabinene	971	4.5
4	ß‐Pinene	985	1.2
5	3‐Octanone	993	2.3
6	Myrcene	998	0.9
7	2‐Caren	1006	0.8
8	α‐Phylandrene	1011	1.6
9	Carvacrol	1310	2.6
10	α‐Fenchen	1016	5.7
11	α‐Terpinene	1027	1.3
12	p‐Cymene	1034	17.5
13	ß‐Phylandrene	1043	1.1
14	Limonene	1050	1.5
15	γ‐Terpinene	1066	16.8
16	Linalool	1152	1.9
17	Terpinene‐4‐ol	1178	3.8
18	Verbenone	1260	1
19	Thymol	1296	23.3
20	Candinol	1338	3.9
21	Hexadecanoeic acid	1370	3.4
	Total		98.5

### 3.2 Characterization of physicochemical properties of nanoemulsion essential oil

The physical properties of nanoemulsion based on *Origanum vulgare* essential oil and *Carum cupticum* essential oil are presented in Table 3. It was discovered that the mean droplet diameter (Z‐averages) of nanoemulsions was produced with Tween 80 (1 w/w%). The best z‐average (d.nm) is 483.4 nm (*Carum cupticum* essential oil+ nano) followed by 470.1 nm (nanochitosan).

**TABLE 3:** Particle size and distribution of different nanoemulsion coatings.

Group	z‐Average (d.nm)	PDI
Chitosan	315.4	0.254
Nanochitosan	470.1	0.295
*Origanum vulgare* essential oil + nano	406.2	0.328
*Carum cupticum* essential oil + nano	483.4	0.469

### 3.3 Antimicrobial activity

Susceptibility testing of both bacteria to the antibiotics tested showed that both strains *Escherichia coli* ATCC 35218 and *Listeria monocytogenes* ATCC 19115 are resistant to Penicillin G. Apart from penicillin G, *E. coli* was sensitive to all other antibiotics tested. However, *L. monocytogenes* was resistant to two other antibiotics, namely tetracycline and gentamicin. These data are presented in Table 4.

**TABLE 4:** Antibiotic susceptibility of *E. coli* and *L. monocytogenes.*

Antibiotics	Disk content (μg/mL)	*E. coli* (ATCC 35218)		*Listeria monocytogenes* (ATCC 19115)	
		Inhibition zone (mm)	Interpretation	Inhibition zone (mm)	Interpretation
Penicillin G	10	‐	R	‐	R
Vancomycin	30	14	S	16	S
Tetracycline	30	15	S	‐	R
Chloramphenicol	30	18	S	19	S
Ciprofloxacin	5	21	S	23	S
Erythromycin	15	23	S	25	S
Gentamicin	30	15	S	‐	R
Trimethoprim	5	16	S	10	I
Streptomycin	10	12	I	16	S
Cephalexin	30	17	S	13	I

The antibacterial activity of *Origanum vulgare* and *Carum cupticum* essential oils on bacterial strains (*E. coli* and *Listeria monocytogenes*) and the minimum inhibitory concentrations are presented in Table 5. The results show that the essential oils were active on both strains. Both extracts were more active on *Escherichia coli* than on *Listeria monotogenes*. The minimum inhibitory concentrations obtained for each extract on the two strains varied according to the type of essential oil.

**TABLE 5:** Essential oils' antibacterial action against pathogenic bacteria.

Essential oils inhibition zone (mm)	Bacterial strains	
	*E. coli* (ATCC 35218)	*Listeria monocytogenes* (ATCC 19115)
*Origanum vulgare*	21 mm	18 mm
*Carum cupticum*	19 mm	17 mm
Negative control (disc saturated with sterile water as a control)	0 mm	0 mm
Positive control (sodium alginate)	11.24 mm	9.84 mm
Values of MIC for essential oils (μL/100 mL)		
*Origanum vulgare*	500 μL/100 mL	750 μL/100 mL
*Carum cupticum*	500 μL/100 mL	750 μL/100 mL

Table 6 presents the results of minimum inhibitory concentration and minimum bactericidal concentration of *Origanum vulgare* emulsion and *Carum cupticum* emulsion against the tested bacteria by microdilution method, it appears that based on the ratio of MBC to MIC, all the preparations have a bactericidal effect as well as antibiotic power on both bacteria.

**TABLE 6:** Antibacterial properties of *Origanum vulgare* emulsion and *Carum cupticum* emulsion against the tested bacteria.

Strains	Essential oil	MIC	MBC
*E. coli* (ATCC 35218)	*Origanum vulgare*	64 mg/mL	128 mg/mL
	*Origanum vulgare* nanoemulsion	16	32
	*Carum cupticum*	128	256
	*Carum cupticum* nanoemulsion	32	64
*Listeria monocytogenes* (ATCC 19115)	*Origanum vulgare*	256 mg/mL	512 mg/mL
	*Origanum vulgare* nanoemulsion	64	128
	*Carum cupticum*	256	512
	*Carum cupticum* nanoemulsion	32	64

From the data in Table 7, it appears that the chitosan and nanochitosan combined with essential oils have an inhibitory activity on the in vitro growth of the *Escherichia coli* strain. On the other hand, only two of these preparations (nanochitosan + *Origanum vulgare* and nanochitosan + *Carum cupticum*) have a good sensitivity to *Listeria monocytogenes*.

**TABLE 7:** Antibacterial activity of chitosan and nanochitosan pathogenic bacteria.

Antibacterial agents	Bacterial strains	
	*E. coli* (ATCC 35218)	*Listeria monocytogenes* (ATCC 19115)
Chitosan/plate (μL/mL)	500	500
MIC (μg/mL)	125	250
Nanochitosan/plate (μL/mL)	500	500
MIC (μg/mL)	32	64
Chitosan and nanochitosan combined with essential oils		
Chitosan+ *Origanum vulgare*	Sensitive	Semi sensitive
Chitosan+ *Carum cupticum*	Sensitive	Semi sensitive
Nanochitosan+ *Origanum vulgare*	Sensitive	Sensitive
Nanochitosan+ *Carum cupticum*	Sensitive	Sensitive

The addition of phenolic compounds to the essential oils of *Origanum vulgare* and *Carum cupticum* showed that the mixture has an inhibitory effect on the bacterial strains tested. These data are presented in Table 8.

**TABLE 8:** Total phenolic content of chitosan film incorporated with essential oils against pathogenic bacteria.

Antibacterial agents	Total phenol content (mg/mL)	*E. coli* (ATCC 35218)	*Listeria monocytogenes* (ATCC 19115)
Chitosan+ *Origanum vulgare*	65.38 mg/mL	16 mm	14 mm
Chitosan+ *Carum cupticum*	89.63 mg/mL	14 mm	11 mm
Chitosan film (control)	0	0	0

The changes in droplet size are shown in Table 9 as a function of storage time for nanoemulsions stored at room temperature. The droplet size increased very rapidly at room temperature during the first 7 days of observation. The growth rate slowed after 7 days, but the size of the nanoemulsions was still within the appropriate range.

**TABLE 9:** Microbial durability test of essential oil and chitosan in 28 days.

Group	Total volatile nitrogen (TVN)	TBA	Thiobarbituric acid (TBA)	Peroxide during the stability	pH	L*	a*	b*	Antioxidant or DPPH test during the stability of 28 days
C	15.55	0.418	0.418	5.83	5.882	75.84	2.57	27.33	
C	15.55	0.413	0.413	5.84	5.878	75.88	2.5	27.33	
C	15.53	0.414	0.414	5.86	5.881	75.88	2.5	27.36	
*Origanum vulgare* 50	15.25	0.354	0.354	5.56	5.799	72.3	2.25	30.56	65.35
50	15.24	0.35	0.35	5.56	5.795	72.33	2.2	30.55	65.33
150	15.23	0.357	0.357	5.56	5.788	72.33	2.22	30.55	65.3
150	15.17	0.345	0.345	5.66	5.773	72.25	2.2	30.62	65.38
250	15.1	0.341	0.341	5.64	5.774	72.2	2.2	30.55	65.4
250	15.12	0.343	0.343	5.66	5.768	72.2	2.25	30.5	65.42
300	15.04	0.366	0.366	5.46	5.781	72.11	2.18	30.52	65.46
300	15.04	0.36	0.36	5.41	5.777	72.11	2.18	30.54	65.5
300	15	0.361	0.361	5.48	5.785	72.11	2.19	30.54	65.52
*Carum cupticum* 50	15.53	0.354	0.354	5.38	5.774	72.23	2.15	34.87	89.57
50	15.52	0.357	0.357	5.34	5.771	72.23	2.23	34.89	89.6
150	15.51	0.351	0.351	5.38	5.768	72.28	2.21	34.89	89.63
150	15.22	0.227	0.227	5.48	5.681	72.15	2.2	35	89.78
250	15.23	0.221	0.221	5.42	5.677	72.13	2.18	34.87	89.77
250	15.23	0.224	0.224	5.46	5.679	72.09	2.2	34.87	89.79
300	15.35	0.213	0.213	5.46	5.671	71.95	2.17	35	89.9
300	15.35	0.213	0.213	5.46	5.671	71.95	2.17	35	89.95
300	15.35	0.213	0.213	5.46	5.671	71.95	2.17	35	89.95
Stability of 7 day									
C	15.76	0.506	0.506	10.21	5.923	73.18	2.55	27.41	
C	15.75	0.502	0.502	10.29	5.918	73.15	2.6	27.48	
C	15.74	0.502	0.502	10.3	5.917	73.2	2.6	27.41	
*Origanum vulgare* 50	15.47	0.416	0.416	7.55	5.811	71.2	2.33	30.66	
50	15.43	0.412	0.412	7.51	5.818	71.21	2.3	30.66	
150	15.45	0.414	0.414	7.59	5.811	71.2	2.3	30.64	
150	15.35	0.408	0.408	6.89	5.797	71.18	2.35	30.68	
250	15.35	0.408	0.408	6.8	5.802	71.2	2.3	30.68	
250	15.35	0.408	0.408	6.85	5.796	71.18	2.3	30.68	
300	15.14	0.413	0.413	7.33	5.812	70.9	2.3	30.59	
300	15.13	0.411	0.411	7.37	5.818	70.95	2.3	30.54	
300	15.12	0.413	0.413	7.4	5.814	70.97	2.33	30.6	
*Carum cupticum* 50	15.55	0.404	0.404	6.97	5.793	71.2	2.4	34.87	
50	15.55	0.401	0.401	6.9	5.793	71.2	2.44	34.88	
150	15.55	0.404	0.404	7.02	5.79	71.2	2.45	34.86	
150	15.3	0.268	0.268	6.34	5.693	71.15	2.5	35	
250	15.33	0.264	0.264	6.37	5.699	71.13	2.5	35.05	
250	15.33	0.268	0.268	6.31	5.697	71.15	2.44	35.07	
300	15.22	0.256	0.256	6.21	5.69	70.9	2.44	35.05	
300	15.21	0.253	0.253	6.2	5.687	70.97	2.4	35.11	
300	15.25	0.256	0.256	6.21	5.685	70.93	2.41	35.08	
Stability of 14 day									
C	16	0.623	0.623	13.65	5.996	72.32	2.42	27.01	
C	15.91	0.622	0.622	13.6	5.994	72.3	2.42	26.99	
C	15.92	0.62	0.62	13.61	6.001	72.31	2.4	26.99	
*Origanum vulgare* 50	15.65	0.525	0.525	8.22	5.833	70.88	2.45	30.72	
50	15.67	0.521	0.521	8.26	5.837	70.87	2.42	30.72	
150	15.61	0.527	0.527	8.24	5.836	70.86	2.45	30.72	
150	15.47	0.514	0.514	7.35	5.811	70.67	2.43	30.81	
250	15.45	0.512	0.512	7.31	5.815	70.67	2.47	30.82	
250	15.5	0.511	0.511	7.39	5.809	70.67	2.45	30.8	
300	15.2	0.513	0.513	9.2	5.827	70.35	2.45	30.63	
300	15.22	0.515	0.515	9.21	5.819	70.33	2.46	30.63	
300	15.2	0.518	0.518	9.24	5.823	70.3	2.44	30.61	
Carum cupticum 50	15.68	0.508	0.508	8.94	5.811	70.85	2.55	34.92	
50	15.67	0.501	0.501	8.95	5.815	70.84	2.52	34.89	
150	15.65	0.503	0.503	8.91	5.807	70.85	2.57	34.9	
150	15.48	0.364	0.364	7.21	5.805	70.65	2.56	35.11	
250	15.45	0.361	0.361	7.22	5.805	70.68	2.59	35.11	
250	15.48	0.36	0.36	7.27	5.805	70.69	2.57	35.12	
300	15.35	0.356	0.356	7.01	5.694	70.33	2.52	35.25	
300	15.33	0.351	0.351	6.93	5.691	70.35	2.58	35.25	
300	15.3	0.357	0.357	6.96	5.694	70.33	2.58	35.23	
Stability of 21 day									
C	16	0.763	0.763	17.44	6.114	70.21	2.54	26.88	
C	15.88	0.768	0.768	17.48	6.118	70.22	2.5	26.85	
C	15.89	0.75	0.75	17.4	6.12	70.24	2.51	26.83	
*Origanum vulgare* 50	15.55	0.661	0.661	11.35	5.854	70.31	2.66	31.36	
50	15.55	0.664	0.664	11.31	5.857	70.3	2.66	31.36	
150	15.55	0.667	0.667	11.38	5.85	70.28	2.66	31.36	
150	15.33	0.651	0.651	10.11	5.848	70.56	2.73	31.55	
250	15.35	0.65	0.65	10.15	5.85	70.6	2.73	31.53	
250	15.32	0.651	0.651	10.19	5.843	70.56	2.73	31.51	
300	15.17	0.631	0.631	12.66	5.844	70.14	2.77	31.77	
300	15.15	0.638	0.638	12.61	5.843	70.16	2.8	31.77	
300	15.15	0.628	0.628	12.63	5.847	70.14	2.82	31.77	
Carum cupticum 50	15.51	0.611	0.611	12.31	5.848	70.73	2.65	35.26	
50	15.53	0.616	0.616	12.31	5.848	70.7	2.68	35.3	
150	15.52	0.62	0.62	12.31	5.85	70.67	2.69	35.28	
150	15.33	0.517	0.517	9.46	5.815	70.63	2.59	35.54	
250	15.35	0.514	0.514	9.5	5.819	70.6	2.63	35.59	
250	15.37	0.516	0.516	9.49	5.82	70.58	2.62	35.57	
300	15.25	0.503	0.503	9.31	5.718	70.31	2.7	35.73	
300	15.22	0.503	0.503	9.3	5.711	70.31	2.71	35.69	
300	15.22	0.503	0.503	9.31	5.711	70.3	2.71	35.71	
Stability of 28 day									
C	16	0.937	0.937	22.57	6.177	70.21	2.42	27.01	
C	15.91	0.938	0.938	22.57	6.18	70.22	2.42	26.99	
C	15.92	0.94	0.94	22.57	6.185	70.24	2.4	26.99	
*Origanum vulgare* 50	15.65	0.661	0.661	15.11	5.868	70.31	2.45	30.72	
50	15.67	0.66	0.66	15.15	5.871	70.3	2.42	30.72	
150	15.61	0.659	0.659	15.13	5.875	70.28	2.45	30.72	
150	15.47	0.661	0.661	14.88	5.869	70.56	2.43	30.81	
250	15.45	0.657	0.657	14.81	5.861	70.6	2.47	30.82	
250	15.5	0.66	0.66	14.87	5.87	70.56	2.45	30.8	
300	15.2	0.631	0.631	15.2	5.888	70.14	2.45	30.63	
300	15.22	0.63	0.63	15.21	5.88	70.16	2.46	30.63	
300	15.2	0.631	0.631	15.27	5.895	70.14	2.44	30.61	
Carum cupticum 50	15.68	0.611	0.611	15.01	5.887	70.73	2.55	34.92	
50	15.67	0.611	0.611	15.09	5.889	70.7	2.52	34.89	
150	15.65	0.611	0.611	15.04	5.894	70.67	2.57	34.9	
150	15.48	0.779	0.779	12.66	5.825	70.63	2.56	35.11	
250	15.45	0.78	0.78	12.63	5.83	70.6	2.59	35.11	
250	15.48	0.781	0.781	12.61	5.828	70.58	2.57	35.12	
300	15.35	0.681	0.681	12.24	5.728	70.31	2.52	35.25	
300	15.33	0.684	0.684	12.21	5.734	70.31	2.58	35.25	
300	15.3	0.678	0.678	12.22	5.73	70.3	2.58	35.23	

## 4 DISCUSSION

The primary goal of this study was to evaluate the antioxidant and antibacterial activity of the sodium alginate nanoemulsion coating incorporated with oregano (*Origanum vulgare* L.) essential oil and *Trachyspermum ammi* (*Carum cupticum*) oil on *Escherichia coli* and *Listeria monocytogenes*. In the food sector, the incursion of nanotechnological advances is still at an embryonic stage but is attracting more and more interest from the scientific and industrial community (Acevedo‐Fani et al., 2015; Salvia‐Trujillo et al., 2015). Recent studies have reported emulsions with small droplets, typically 10–100 nm in size (nanoemulsions) being used as lipophilic drug delivery systems in food, cosmetics, and pharmaceutical products. Due to their intrinsic properties, these nanoemulsions can have several advantages for the encapsulation of functional lipophilic compounds compared to conventional emulsions. Moreover, reducing the size of the droplets, in addition to improving the transport of active molecules across biological membranes, could also increase the surface‐to‐volume ratio, thus leading to better functionality of the products (Acevedo‐Fani et al., 2015; Salvia‐Trujillo et al., 2015).

The GC–MS analysis of the volatile constituents of the *Origanum vulgare* essential oil compounds allowed the identification of 19 compounds representing 93.72% of the total oil. The major components detected in *Origanum vulgare* essential oil were pulegone (49.25%), eucalyptol (18.23%), and menthone (12.37%). About the essential oil of *Carum cupticum*, it has been identified 21 compounds representing 98.5% of the total oil. The main components detected in the essential oil of *Origanum vulgare* were thymol (23.3%), p‐cymene (17.5%), and γ‐terpinene (16.8%). The other components identified were minor, ranging from 1% to 5.7%. From all these data relating to the chemical composition of the essential oil of the plants studied, it could be deduced that these plants are rich in bioactive composition. These data are consistent with other literature data (Caputo et al., 2017; Snuossi et al., 2016). Another prior study of Wang et al. (2019) indicated that the essential oil of these plants contained less compound. The differences obtained in these studies could be justified by the consequence of several factors including, among others, the geographical location of the plants, the environmental conditions, the age of the plant, the stage of development, the harvest period, the chemical composition of the soil and the extraction method used in the studies. As such, it is important to remember that these studies were not carried out in the same area and few data exist regarding the method of extraction of essential oils.

The physical properties of nanoemulsion based on *Origanum vulgare* and *Carum cupticum* essential oils respectively show mean droplet diameters (Z‐averages) of nanoemulsions prepared with Tween 80 (1% by weight). The best Z‐average (in nm) is 483.4 nm (*Carum cupticum* essential oil + nano), followed by 470.1 nm (nanochitosan). In general, the diameter of nanoemulsions may be affected by surfactant concentration, which is indirectly proportional to the droplet size's external exposure. The oil/water interfacial tension may also play a role (Sundararajan et al., 2018). It is also affected by the shear forces and turbulence produced by the ultrasonic homogenizer (Mehmood et al., 2017). The comparison of the data from this study with other research works in the literature, it was demonstrated that the nanoemulsion based on the essential oils tested favored the increase in its droplet size (247.52 nm) compared to that found in the study carried out by Ozogul et al. (2017) (66.02 nm). Also, according to the Tween 80, the concentrations decreased by 3–1% (w/w). On the other hand, the application of Tween 80 as nonionic surfactants in the preparation of oil‐in‐water emulsions has been favorable due to its hydrophilic–lipophilic balance (HLB) (Chu et al., 2020). Furthermore, Tween 80, as a small molecule surfactant, was more effective in adsorbing on the droplet surface when compared to polymer‐based surfactants (Chu et al., 2020).

Previous studies have demonstrated the efficacy of nanoemulsions in improving the bioavailability and stability of essential oils, resulting in enhanced antimicrobial activity (Liu et al., 2020). From this research study, the antimicrobial activities of nanoemulsion‐based *Origanum vulgare* essential oil and *Carum cupticum* essential oil and pure essential oil of these plants against food‐borne pathogens were examined qualitatively and quantitatively using disk diffusion test, followed by MIC and MBC assays. The results show that the different preparations have a good inhibitory activity for the in vitro growth of *Escherichia coli* and *Listeria monocytogenes*. The study of Fournomiti et al. (2015) showed that the essential oils from these plants have good inhibitory activity on *Klebsiella pneumonia*, *Escherichia coli*, and *Enterobacter cloacae*. Also, found that *P. aeruginosa* demonstrated the highest resistance, while *B. cereus* was the most sensitive to the oregano oil with (MIC) ranging from 1.56 to 50 μL/mL (Jnaid et al., 2016). Recently in another study, oregano essential oil at a concentration of 0.02% (v/v) demonstrated antibacterial and antifungal activity, with total elimination of all the tested strains until the sixth day of ripening, besides inhibiting the germination of fungal spores (Leonelli Pires de Campos et al., 2022).

Indeed, the phytochemical composition of the essential oil of these two plants would have had the ability to interact with the lipids of the bacterial cell membrane, thus disrupting the fundamental cellular structures by interrupting the conditions of homeostasis of the microbial cell. Plants could also affect bacteria by other mechanisms such as destruction of the bacterial wall, inhibition of replication of bacterial genetic material and many others. Thus, these substances could lead to the death of bacterial cells due to the escape of its cellular content, the inability of replication and the absence of the protective action of the bacterial wall (Yazgan et al., 2019). Its most important antibacterial mechanisms are enzyme inhibition, efflux pump inhibition, ATP depletion, biofilm formation inhibition, and cytoplasmic membrane damage (Soltani et al., 2021). Although NE can be seen as an effective means for EOs to improve their physicochemical stability and reduce their volatility, it is also possible that it interacts with microbial cells, which could lead to their destruction by following (de Matos et al., 2019; Yazgan et al., 2019). According to the MIC and MBC results of this study, the nanoemulsion also presented a good bacteriostatic activity against the two pathogenic bacteria tested in this study. In addition, the susceptibility testing of both bacteria to the antibiotics tested showed that both strains (*Escherichia coli* ATCC 35218 and *Listeria monocytogenes* ATCC 19115) are resistant to Penicillin G. Apart from penicillin G, *E. coli* was sensitive to all other antibiotics tested. However, *L. monocytogenes* was resistant to two other antibiotics, namely tetracycline and gentamicin. In general, nanoemulsion‐based delivery methods of terpene oils or essential oil show excellent antimicrobial activity of encapsulated bioactive compounds. Indeed, terpene oils in nanoparticles could, in fact, acquire novel and biological features such as biodegradability, permeability, thermal stability, solubility, rigidity, and crystallinity (Almadiy et al., 2016).

## 5 CONCLUSION

In conclusion, the nanoemulsion‐based essential oils derived from *O. vulgare* and *C. cupticum*, as investigated in this study, demonstrated remarkable richness in phytochemical components. The antioxidant potential observed in these nanoemulsion formulations highlights the synergistic effects of nanoemulsions in enhancing the bioavailability and efficacy of essential oil components. This study also provides valuable insights into the antimicrobial potential of nanoemulsion‐based essential oils from *O. vulgare* and *C. cupticum* against *E. coli* and *L. monocytogenes*. The enhanced inhibitory and bacteriostatic activities observed in the nanoemulsion formulations underscore the potential applications of nanotechnology in improving the efficacy of essential oils as natural antimicrobial agents. These findings not only contribute to our understanding of the antioxidant properties inherent in nanoemulsion‐based essential oils from *O. vulgare* and *C. cupticum* but also underscore their potential applications in functional foods and pharmaceuticals, where antioxidant activities are highly valued for promoting health and preventing oxidative stress‐related disorders. Further research is warranted to explore the practical applications and optimization of these formulations in food safety and preservation. The use of nanoemulsions as carriers for essential oils from *O. vulgare* and *C. cupticum* presents a promising avenue for the development of effective antimicrobial agents in the food industry.

## AUTHOR CONTRIBUTIONS

**Hashem Saffarian:** Formal analysis (equal); investigation (equal); methodology (equal); writing – original draft (equal). **Ebrahim Rahimi:** Project administration (equal); supervision (equal); validation (equal); visualization (equal); writing – review and editing (equal). **Faham Khamesipour:** Conceptualization (equal); writing – review and editing (equal). **Seyed Majid Hashemi Dehkordi:** Visualization (equal); writing – review and editing (equal).

## FUNDING INFORMATION

The authors state that they have no known competing financial interests or personal interests that could have seemed to affect the work reported in this paper.

## CONFLICT OF INTEREST STATEMENT

The authors state that they have no known competing financial interests or personal relationships that could have seemed to affect the work reported in this paper.

## Data Availability

Because no new data were created or processed in this study, data sharing does not apply to this paper.
